# Effect of Vitamin D Replacement on Cognition in Multiple Sclerosis Patients

**DOI:** 10.1038/srep45926

**Published:** 2017-04-04

**Authors:** Hala Darwish, Ribal Haddad, Sahar Osman, Stephanie Ghassan, Bassem Yamout, Hani Tamim, Samia Khoury

**Affiliations:** 1 American University of Beirut, Faculty of Medicine, Hariri School of Nursing, Beirut, Lebanon; 2 American University of Beirut Medical Center, Nehme and Therese Tohme Multiple Sclerosis Center, Beirut, Lebanon; 3 American University of Beirut, Faculty of Medicine, Neurology Department, Beirut, Lebanon; 4 American University of Beirut, Faculty of Medicine, Department of Internal Medicine and Clinical Research Institute, Beirut, Lebanon

## Abstract

Multiple Sclerosis is associated with deficient serum 25 hydroxyvitamin D (25 (OH)D) level and cognitive impairment. The aim of this study is to evaluate cognitive performance in MS patients with deficient 25 (OH)D (<25 ng/ml) compared to patients with sufficient levels (>35 ng/ml), then to evaluate the change in cognitive performance after 3 months of vitamin D3 oral replacement. Eighty-eight MS patients with relapsing remitting and clinically isolated type of MS, older than 18 years treated with interferon beta were enrolled. Cognitive testing was performed at baseline and at 3 months using the Montreal Cognitive Assessment (MoCA), Stroop, Symbol Digit Modalities (SDMT) and Brief Visuospatial Memory Test (BVMT-R). Serum 25 (OH)D was measured at baseline and at the end of the study. Vitamin D3 replacement improved the MS patients’ cognitive performance after 3 months on the MoCA and BVMT-Delayed Recall (DR). Sufficient serum 25 (OH)D level predicted better cognitive performance on the BVMT-DR at baseline (β: 1.74, p: <0.008) and 3 months (β: 1.93, p: <0.01) after adjusting for all measured confounding variables. Vitamin D_3_ replacement could improve cognitive performance in MS patients and make a significant difference in the patient’s quality of life.

Multiple Sclerosis (MS) is a chronic inflammatory disease of the central nervous system that is linked to environmental factors such as smoking and vitamin D level. Low serum 25 hydroxyvitamin D (25 (OH) D) is associated with increased risk of MS^1^,^2^ and increased disease activity in patients with MS^3^,^4^. Vitamin D supplementation protects from disease in MS animal models^5^,^6^,^7^. Furthermore, Vitamin D levels appear to be lower in MS patients than controls^8^,^9^.

Cognitive impairment is a common symptom of MS that impacts quality of life, and affects up to 65% of patients^10^,^11^,^12^. The impairment affects a wide range of cognitive functions including verbal and non-verbal memory, and information processing speed^12^. Studies in humans provide support for a role of the vitamin D in cognitive functioning, and recent studies have linked low serum 25 (OH)D levels to cognitive dysfunction in adults^13^,^14^,^15^. Vitamin D receptors are expressed by neurons and glial cells^16^,^17^ supporting a role for vitamin D beyond bone homeostasis. Based on prevalence of hypovitaminosis D in our clinical population^9^, we hypothesized that low serum 25 (OH)D has a negative effect on cognitive performance. This study aim was to measure cognitive performance in patients with serum 25 (OH)D deficiency (<25 ng/ml) compared to patients with sufficient levels (>35 ng/ml), then evaluate the change in cognitive performance in 25 (OH)D deficient subjects after supplementation with oral vitamin D_3_ (Cholecalciferol, Euro-D 10000, Montreal, Quebec, Canada).

## Results

**Figure 1:**
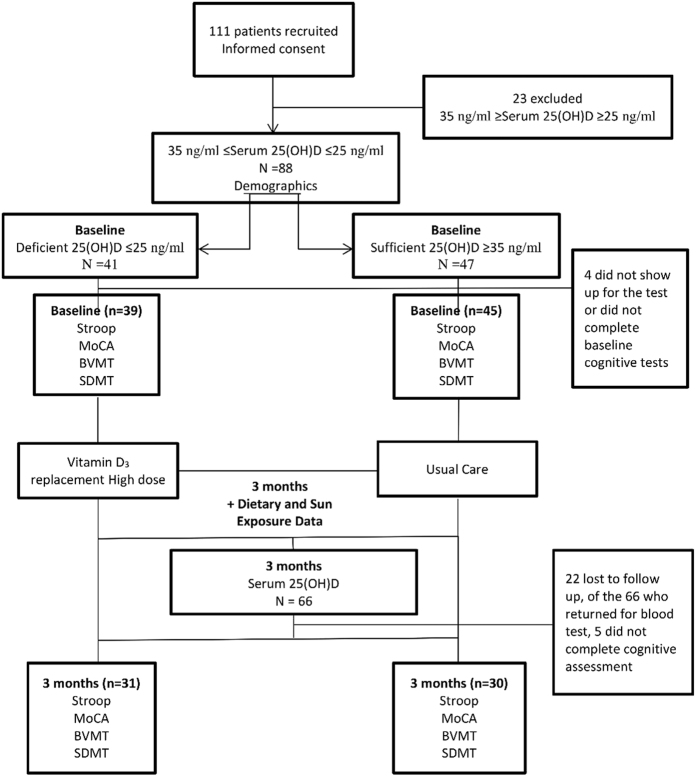
Flow Diagram of the subjects enrollment from recruitment through study completion.

A total of 111 participants were recruited, of whom 23 had 25 (OH)D levels between 25 and 35 ng/ml (inclusive) and were thus excluded from the study. Of the remaining 88 participants, 40 were males (45.4%) and the average age of all the participants was 36.3 ± 12.2 years. At baseline, there were 47 (53.4%) patients with sufficient and 41 (46.6%) with deficient 25 (OH)D. Of those, 84 participants underwent cognitive tests (39 in deficient group and 45 in sufficient group). After 3 months, 22 patients were lost to follow up; 66 had their serum 25 (OH)D level tested; 61 of them completed the study and underwent cognitive tests (31started in deficient group and 30 in sufficient group) (Fig. 1 and Table 1).

### Serum 25 (OH)D

Mean 25 (OH)D serum level was 15.8 ± 6.5 ng/ml in the deficient group and 59.6 ± 24.5 ng/ml in 25 (OH)D sufficient group (P < 0.0001). After supplementation with high dose vitamin D_3_ for three months, 25 (OH)D level increased to 49.0 ± 14.6 ng/ml (p < 0.001 compared to baseline) in the deficient group, although it remained lower than the sufficient group that received usual care 64.2 ± 18.7 ng/ml (p < 0.001).

Despite weekly phone calls to remind participants to complete their diaries, only 30 of 88 (34%) participants completed it. There was no statistically significant difference between groups in terms of sun exposure (2.22 ± 2.08 vs. 2.16 ± 1.57 hours/week, p = 0.79) number of body parts exposed to the sun (1.29 ± 1.27 vs. 1.42 ± 0.84, p = 0.46), estimated weekly vitamin D intake from rich food sources; such as, fatty fish and egg yolks (653.17 ± 509.89 vs. 736.41 ± 582.09 IU/week, p = 0.676) and body mass index (26.1 ± 4.23 vs. 24.8 ± 2.9, p = 0.11).

### Sample characteristics per group

There were no significant differences between the two groups in age, gender, disease duration, and other demographic factors (Table 1). However, patients with deficient 25 (OH)D level had higher EDSS scores (1.6 ± 1.1 vs. 1.1 ± 0.9, p = 0.04) and reported significantly less engagement in physical activity than those with sufficient 25 (OH)D (38.9% vs. 65%, p = 0.02), and were involved in more leisure activities such as reading, watching TV and playing board games than those with sufficient 25 (OH)D; but the difference did not reach statistical significance (0.243 ± 0.271 vs. 0.152 ± 0.189, p = 0.07). The anxiety score was significantly lower in subjects with sufficient 25 (OH)D than the 25 (OH)D deficient group (1.7 ± 0.6 vs. 2.1 ± 0.7, p = 0.008). Total depression and anxiety score tended to be higher in the deficient 25 (OH)D compared to sufficient group; however, this was not statistically significant (2.1 ± 0.7 vs. 1.8 ± 0.6, p = 0.07).

### Cognitive performance after vitamin D_3_ replacement per group

The cognitive performance of the group with deficient 25 (OH)D group improved significantly on the, BVMT delayed recall (p = 0.02) and the MoCA (p = 0.006) after 3 months of vitamin D_3_ replacement; but not on the SDMT. A composite score of the BVMT learning trials (T1–T3) was computed; both groups showed improvement on the total BVMT trials that was statistically significant (p = 0.003 for the sufficient; and p = 0.004 for the deficient). Moreover, both groups showed improvement on the Stroop test; however, it was not statistically significant (p = 0.56) (Table 2). The SDMT scores adjusted for age and years of education of the majority of participants regardless of their 25 (OH)D level was within normal limits [100% of the deficient 25 (OH)D and 94.3% of the sufficient group] at baseline. Similarly, the majority of the sample showed no interference effect on Stroop test indicating intact attention [69.2% of the deficient 25 (OH)D and 60% of the sufficient group].

#### Multivariate analysis

To further explore the vitamin D_3_ replacement effect on cognitive performance, after adjusting for other confounding variables such as age and years of education, a multivariate analysis was performed for all outcome variables at baseline and at three months for the: Stroop, MoCA, SDMT and BVMT-R (T1, T2, T3, Total score and DR). Dependent variables included in the model were: baseline 25 (OH)D status (sufficient vs. deficient), at 3 months 25 (OH)D status (sufficient vs. deficient), disease duration, EDSS, age, education (college or high school), physical activity, smoking, alcohol, score of leisure activities, anxiety score, depression score, and total Hopkins score. The results showed that baseline 25 (OH)D status was a significant predictor of performance on the BVMT delayed recall both at baseline and at three months after controlling for all other variables. Those with deficient 25 (OH)D level at baseline were found to have 1.74 (95% CI [−3.01, 0.47], p = 0.008) and 1.93 (95% CI [−3.42, −0.44], p = 0.01) points less on BVMT-DR compared to those with sufficient level, at baseline and at 3 months respectively. It was also positively correlated with immediate recall at three months on the BVMT-T2 (10 seconds immediate recall) (95% CI [−3.18, −0.2], p = 0.03), but not significant for the total BVMT trials 1–3 score at baseline, and at 3 months (Tables 3 and 4).

As expected, age was inversely correlated (at baseline and after 3 months) with the Stroop, SDMT, BVMT-T3 and BVMT-DR. Age also correlated inversely at baseline with MoCA and BVMT-T1 (Tables 3 and 4). The anxiety score was inversely correlated with Stroop at the second, SDMT and MoCA at baseline (Tables 3 and 4). EDSS was negatively correlated with the total BVMT score at baseline and follow up, and with the MoCA, and BVMT-T1 at the follow up visit. High school and college education were positively correlated at baseline with Stroop, and college education with the total BVMT score at baseline and at 3 months. College education was also positively correlated with the BVMT-T1, and correlated positively after three months with SDMT and MoCA (Tables 3 and 4). Leisure score correlated positively with BVMT-T2 at both visits; whereas, it correlated positively with the BVMT-total Score and DR at the second visit. Alcohol intake positively correlated with the BVMT-total score at 3 months and with the SDMT at both visits; and BVMT-T1 at the second visit (Tables 3 and 4).

## Discussion

There is a known association between 25 (OH)D levels and cognitive performance^15^,^18^,^19^ in older adults, but the relationship between 25 (OH)D and cognition in MS patients has not been explored. In this report, we show that a relatively short course of oral supplementation with vitamin D_3_ in MS patients deficient in serum 25 (OH)D, improved memory as measured by the BVMT Delayed Recall and MoCA (general memory test that includes long term as well as visuo-spatial memory). Moreover, a level of 25 (OH)D >35 ng/ml was a predictor of a better cognitive performance on the BVMT-DR after adjusting for all measured confounding variables.

The Stroop and SDMT are sensitive to processing speed and attention and can be impaired in MS; studies showed significant SDMT changes over 5–10 years and mostly in progressive MS subtypes^20^,^21^,^22^. Not surprisingly given their short disease duration, the participants in this study had normal performance of the STROOP and SDMT. As processing speed is predictive of performance on visuospatial learning and memory tests^23^, this may explain our findings of improvement on the BVMT-T1-3 total score in both groups at 3-months (practice effect). In this study we uncovered an interesting uncoupling between performance on immediate and delayed recall in the subjects with 25 (OH)D deficiency. Given that delayed recall is a more demanding process this could indicate that the effect of 25 (OH)D is more important for long-term, demanding, memory. Interestingly, hippocampal neurons (necessary for delayed retrieval processes) express vitamin D receptors^17^.

EDSS and low physical activity were significantly higher in the low 25 (OH)D group. With a greater disease severity as measured by EDSS, which places a great emphasis on mobility and walking, participants are less likely to exit their homes and get sun exposure. Moreover, low 25 (OH)D levels correlate with greater disability and relapse rate in multiple sclerosis^7^. Further, subjects in our study who engaged in non–physical leisure activities were more likely to have lower 25 (OH)D as well; possibly as a consequence of a higher EDSS score and decreased physical activity, leading them to stay inside and engage in board games, reading, or watching television, among others.

Similarly, anxiety scores were significantly higher in the low 25 (OH)D group compared to the sufficient 25 (OH)D group. This is consistent with previous studies in rats and humans that demonstrated that 25 (OH)D plays an integral role in anxiety centers in the brain^24^,^25^.

The main limitation of this study is the short time frame. Three months was chosen to assess the earliest possible effect of vitamin D_3_ replacement on cognitive performance in MS; however, three months may not have been sufficient for a true improvement in cognition to be observed between groups. Several previous studies have shown delayed improvement in cognition with stroke^26^. A study with donezepil demonstrated improvement in cognition after 24 weeks^27^,^28^ while most studies of cognitive rehabilitation in multiple sclerosis patients found improvement at 6 months^29^. Moreover, further studies to assess the effect of vitamin D_3_ replacement on subjects with higher EDSS scores and longer disease duration are also needed. It would be important to test whether long-term vitamin D_3_ supplementation along with other cognitive rehabilitation interventions can reverse cognitive deficits.

Another possible limitation of this study is the poor response rate to lifestyle diary, not allowing true control for other sources of vitamin D as possible confounders, such as sun exposure and dietary vitamin D. However, there was no difference between groups in terms of sun exposure, vitamin D intake and body mass index among the 30 patients who completed their diaries.

In summary, our study suggests a positive effect of vitamin D_3_ supplementation on cognitive function in MS. Thus, exploring the longitudinal effect (at least 1 year) of vitamin D_3_ replacement on cognitive performance in a larger sample of MS subjects with 25 (OH)D deficiency is being planned; while placing more emphasis on additional memory components; such as, verbal memory. Improving cognition early in the course of the disease with a simple intervention such as vitamin D_3_ supplementation could make a significant difference in the patient’s quality of life.

## Methods

### Participants and Study design

This prospective study was conducted at the MS center of the American University of Beirut Medical Center over a span of two years (2012–2014). A sample size of 88 was calculated for the primary outcome of immune marker changes, the results of this study will be reported separately. A Post-hoc analysis on the BVMT-DR results on the available subjects showed a power of 0.74.

Eighty-Eight participants MS patients were diagnosed according to McDonald 2010 criteria^30^. Males and females aged 18 or older with Relapsing Remitting MS or Clinically Isolated Syndrome, clinically stable, in other words no exacerbations (defined as an episode of neurologic dysfunction lasting at least 24 hours) within four weeks of enrollment, on interferon beta with no gadolinium enhancing lesions on MRI and did not receive any corticosteroid therapy within four weeks prior to recruitment were enrolled. MRI of brain and spinal cord with and without gadolinium were obtained as part of their usual care. All patients using drugs associated with hypercalcemia or treated with disease modifying therapies other than IFN-b within six weeks prior to enrolment, pregnant and with history of primary hyper PTH, hypercalcemia, renal dysfunction, cardiac disease, malignancy, granulomatous disease, dementia, traumatic brain injury, diagnosis of epilepsy or history of seizure, psychiatric disease, substance abuse/dependence, alcohol abuse/dependence were excluded from the study. Patients with 25 (OH)D serum level between 25 and 35 ng/ml were also excluded.

Subjects were recruited and assigned to sufficient (25 (OH)D >35 ng/ml) and deficient (<25 ng/ml) 25 (OH)D groups. Subjects with deficient 25 (OH)D levels were given a high dose vitamin D_3_ supplementation (10,000 IU daily for 3 months). Subjects with sufficient (25 (OH)D >35 ng/ml) continued their usual treatment that may include vitamin D_3_ supplementation at various doses. Cognitive tests were performed at baseline and at 3 months.

### Standard protocol approvals, registrations, and patient consents

This study was registered in 19/09/2013 at clinical Trials.gov with the identification number NCT01952483. The study protocol is available in the supplementary information. The American University of Beirut Institutional Review Board approved this study, protocol number IM.SK1.04. All methods were performed in accordance with the guidelines and regulations of the American University of Beirut Institutional Research Board and all participants signed an informed consent.

### Screening and other assessment tools

*Hopkins Symptoms Checklist (HSCL-25*) was administered at baseline to screen all participants for any depressive or anxiety symptoms, which may be a confounding factor on cognitive function. This is a 25-item tool that has been translated into Arabic and is widely used due to its brevity, reliability, and validity^18^,^19^,^20^,^21^,^22^,^23^,^24^,^25^,^26^,^27^,^28^,^29^,^30^,^31^.

#### Brief Risk Factor Surveillance System (BRFSS)

Excerpts from BRFSS^32^ were filled out to collect demographic data such as age, years of education, and lifestyle factors known to affect cognitive function such as smoking, exercise, alcohol intake and recreational habits. All medications (including anti-depressants or anxiolytics) were recorded at each visit.

#### Food Diaries

To control for additional vitamin D intake from food sources, all subjects were handed a diary to document their daily ingestion of food known to be rich in vitamin D such as salmon, egg yolk and to document their body parts exposure to sun as well. A research assistant contacted each participant weekly to collect information on their food intake, ensure compliance with their supplementation and that they are filling their diaries which were collected at the end of the three months period (Fig. 1).

#### Cognitive assessment

A cognitive test battery measuring different fields was administered. The test took around 45 minutes to complete (Table 5).

*Montreal Cognitive Assessment* (MoCA-10 minutes) is a screening tool for cognitive impairment in adults. The MoCA-Arabic version has demonstrated 92.3% sensitivity and 85.7% specificity for detecting mild cognitive impairment in older adults in Egypt^33^. We have used this test in a sample of healthy adults and older adults in Lebanon and the test showed high internal consistency (α = 0.83)^15^.

*The Symbol Digit Modalities Test* (SDMT-5 minutes) is a known and widely used speed of processing test. Oral SDMT was administered; it does not require translation and has been used in Lebanon before^15^.

*Stroop test* (Arabic-5 minutes) is a highly established test of attention and was administered based on an earlier description^34^. Subject is given a list of colors in black ink to read (Stroop 1), then a list of colors in their corresponding ink color (Stroop 2), and then finally a list of colors with incongruent ink colors (Stroop 3), the participant is expected to read the word without being affected by interfering mismatching color. The number of words read in a minute (minus the errors) was the dependent measure and interference was calculated using the following equation: Interference = Stroop 3 − [(Stroop 1 + Stroop 2)/2].

*Brief Visuospatial Memory test* (BVMT-R- 25 minutes) is a visuospatial memory test that is comprised of three memory trials (10 seconds each) followed by delayed recall after 25 minutes and a recognition trial. It has been widely used as a quick measure of memory at bedside and in MS patients^35^.

#### Serum 25 (OH)D testing

Serum 25 (OH)D levels were measured in the Endocrine Core laboratory at AUBMC using Roche Diagnostics total assay. This assay has a between day precision of CV = 9.4% and 1.9% at mean concentrations of 43.3 and 105 nmol/L using controls as provided by Roche Diagnostics as well as a mean percent bias of less than 5% from all laboratory trimmed mean when compared to IDS RIA or IDS iSYS assays^36^.

### Statistical analysis

#### Predictor variables

25 (OH)D status (sufficient Y/N), –25 (OH)D, age, years of education, physical activity, sun exposure, dietary vitamin D intake, smoking, leisure activity and alcohol consumption –were analyzed using descriptive measures. Independent sample and paired *t*-tests (before and after and between groups) and Pearson’s *r* correlations were performed between predictor and outcome variables, MoCA, Stroop, BVMT-Trials 1 to 3 and total score, BVMT-DR and SDMT. A stepwise linear regression analysis was conducted to examine the relationship between predictor and outcome variables. Only complete cases were used in the analysis.

## Additional Information

**How to cite this article:** Darwish, H. *et al*. Effect of Vitamin D Replacement on Cognition in Multiple Sclerosis Patients. *Sci. Rep.* **7**, 45926; doi: 10.1038/srep45926 (2017).

**Publisher's note:** Springer Nature remains neutral with regard to jurisdictional claims in published maps and institutional affiliations.

## Supplementary Material

Supplementary Information

## Figures and Tables

**Table 1:** Sample characteristics per group.

			Sufficient 25 (OH)D N (%)	Deficient 25 (OH)D N (%)	P value
			N = 47	N = 41	
**Age**	Mean (±sd)		37.2 (±12.3)	35.3 (±12.3)	0.48
**BMI**	Mean (±sd)		26.1 (±4.23)	24.8 (±2.9)	0. 11
**Gender**	Male		20 (42.6%)	20 (48.8%)	0.56
	Female		27 (57.4%)	21 (51.2%)	
**Living situation**	Alone		2 (5.4%)	3 (8.6%)	0.60
	With others		35 (94.6%)	32 (91.4%)	
Disease duration (years)	Mean (±sd)		5.7 (±6.5)	4.8 (±6.4)	0.53
**EDSS**	**Mean (**±**sd)**		**1.1 (**±**0.9)**	**1.6 (**±**1.1)**	**0.04**
**Anxiety score**	**Mean (**±**sd)**		**1.7 (**±**0.6)**	**2.1 (**±**0.7)**	**0.008**
Depression score	Mean (±sd)		1.9 (±0.7)	2.1 (±1.0)	0.18
Total Hopkins score	Mean (±sd)		1.8 (±0.6)	2.1 (±0.8)	0.07
Calcium Visit 1	Mean (±sd)		9.7 (±0.4)	9.6 (±0.4)	0.66
Calcium Visit 2	Mean (±sd)		12.4 (±15.9)	9.7 (±0.4)	0.34
**Education level**	Elementary		2 (5.3%)	3 (8.6%)	0.31
	High school		8 (21.1%)	3 (8.6%)	
	College		28 (73.7%)	29 (82.9%)	
**Employment**	Employed for wages		21 (53.8%)	19 (54.3%)	0.28
	Self-employed		6 (15.4%)	4 (11.4%)	
	Out of work for more than 1 year		3 (7.7%)	0 (0.0%)	
	Out of work for less than 1 year		0 (0.0%)	3 (8.6%)	
	Homemaker		5 (12.8%)	4 (11.4%)	
	Student		4 (10.3%)	4 (11.4%)	
	Retired		0 (0.0%)	1 (2.9%)	
**Lifestyle**	**Physical activity**	**No**	**14 (35.0%)**	**22 (61.1%)**	**0.02**
		**Yes**	**26 (65.0%)**	**14 (38.9%)**	
	Disability	No	27 (93.1%)	23 (85.2%)	0.41
		Yes	2 (6.9%)	4 (14.8%)	
	*Smoking cigarettes*	*No*	*24 (61.5%*)	*29 (80.6%*)	*0.07*
		*Yes*	*15 (38.5%*)	*7 (19.4%*)	
	smoking waterpipe	No	25 (69.4%)	19 (65.5%)	0.74
		Yes	11 (30.6%)	10 (34.5%)	
	smoking cigars	No	32 (94.1%)	28 (96.6%)	1.00
		Yes	2 (5.9%)	1 (3.45)	
	*alcohol*	*No*	*23 (60.5%*)	*27 (79.4%*)	*0.08*
		*Yes*	*15 (39.5%*)	*7 (20.65*)	
**Leisure and activities**	Read	No	8 (50.0%)	10 (50.0%)	1.00
		Yes	8 (50.0%)	10 (50.0%)	
	Watch TV	No	7 (31.8%)	7 (35.0%)	0.83
		Yes	15 (68.2%)	13 (65.0%)	
	Video games	No	13 (76.5%)	13 (68.4%)	0.72
		Yes	4 (23.5%)	6 (31.6%)	
	Board games	No	14 (93.3%)	15 (88.2%)	1.00
		Yes	1 (6.7%)	2 (11.8%)	
	Social media	No	9 (47.4%)	10 (50.0%)	0.87
		Yes	10 (52.6%)	10 (50.05)	
	Internet	No	10 (55.6%)	8 (42.1%)	0.41
		Yes	8 (44.4%)	11 (57.9%)	
	Volunteer	No	30 (81.1%)	24 (68.6%)	0.22
		Yes	7 (18.9%)	11 (31.4%)	
	Religious	Religious	18 (50.0%)	19 (65.5%)	0.26
		Spiritual	5 (13.9%)	1 (3.4%)	
		Neither	13 (36.1%)	9 (31.0%)	
	***Score of leisure activities***	***Mean***(±***sd***)	***0.152***(±***0.189***)	***0.243***(±***0.271***)	***0.075***
**Sleep**	Hours of sleep/night	1–3 hours	0 (0.0%)	1 (2.9%)	0.57
		4–7 hours	24 (63.2%)	22 (62.9%)	
		8+ hours	14 (36.8%)	12 (34.3%)	
	Days of sleep insufficiency	1–3 days	9 (25.7%)	8 (32.0%)	0.86
		4–7 days	7 (20.0%)	6 (24.0%)	
		8–12 days	11 (31.4%)	6 (24.0%)	
		13+ days	7 (20.0%)	5 (20.0%)	
		None	1 (2.9%)	0 (0.0%)	
**Memory**	Memory loss/ confusion last year	No	21 (72.4%)	14 (60.9%)	0.38
		Yes	8 (27.6%)	9 (39.1%)	
		Yes	0 (0.0%)	0 (0.0%)	

**Table 2:** Paired t-tests by cognitive test per group.

	Sufficient 25 (OH)D group N=29			Deficient 25 (OH)D group N=31		
	Mean (±sd)	95% (CI)	P value	Mean (±sd)	95% (CI)	P value
STROOP (Baseline)	3.66 (±7.86)	(−7.21, 2.4)	0.06	5.6 (9.2)	(−16.55, 2.6)	0.56
STROOP (3 months)	7.1 (±8.95)			6.6 (11.5)		
SDMT (Baseline)	59.4 (±13.2)	(−4.51, 2.43)	0.53	53.0 (16.9)	(−12.3, 12.8)	0.11
SDMT (3 months)	58.4 (±14.6)			50.2 (12.3)		
BVMT Trial 1 (Baseline)	5.3 (±3.2)	**(−2.30, −2.4)**	**0.02**	4.7 (3.0)	**(−1.77, −1.6)**	**0.02**
BVMT Trial 1 (3 months)	6.6 (±2.6)			5.7 (3.2)		
BVMT Trial 2 (Baseline)	7.8 (±3.4)	**(−2.18, −0.5)**	**0.003**	7.0 (3.8)	(−1.91, 0.10)	0.08
BVMT Trial 2 (3 months)	9.2 (±2.3)			7.9 (3.8)		
BVMT Trial 3 (Baseline)	9.0 (±3.4)	(−1.5, 0.05)	0.07	7.7 (4.1)	**(−2.17,** −**3.4)**	**0.009**
BVMT Trial 3 (3 months)	9.7 (±2.3)			8.9 (3.5)		
BVMT-Trials Composite Score (Baseline)	22.10 (± 9.28)	(−5.52, −1.23)	0.003	19.41 (±10.12)	(−5.17, −1.08)	0.004
BVMT-Trials Composite Score (3 months)	25.48 (±6.6)			22.54 (± 9.75)		
BVMT Delayed Recall (Baseline)	9.4 (±3.0)	(−1.07, 0.10)	0.11	7.5 (3.8)	**(−1.84, 0.16)**	**0.02**
BVMT Delayed Recall (3 months)	9.9 (±2.6)			8.5 (3.8)		
MOCA (Baseline)	26.1 (±2.9)	(−1.47, 0.98)	0.08	24.5 (3.8)	**(−2.7, −5.10)**	**0.006**
MOCA (3 months)	26.8 (±2.8)			26.1 (3.8)		

MoCA = Montreal Cognitive Assessment; SDMT = Symbol Digit Modalities Test; BVMT-R = Brief Visuospatial Memory test. CI = Confidence Interval.

**Table 3:** Multivariate analysis for the predictors of the 7 cognitive performance tests at baseline (stepwise method).

Predictors	STROOP			
	R^2^	Unstandardized Beta	95% CI	P value
25 (OH)D (Sufficient Y/N)	0.20	2.4	−1.39: 6.19	0.21
Education: Elementary		**REF**	**REF**	**REF**
**Education: College**		**12.1**	**4.83: 19.32**	**0.001**
**Education high school**		**9.58**	**1.09: 18.08**	**0.03**
**Age**		−**0.18**	−**0.33:** −**0.03**	**0.02**
**SDMT**				
25 (OH)D (Sufficient Y/N)	0.47	−4.03	−9.08: 1.02	0.12
**Age**		−**0.63**	−**0.83:** −**0.43**	**<0.0001**
**Anxiety score**		−**5.58**	−**9.60:** −**1.56**	**0.007**
**Alcohol**		**6.16**	**0.72: 11.60**	**0.03**
**BVMT T1**				
25 (OH)D (Sufficient Y/N)	0.30	−0.6	−1.68: 0.57	0.33
**Age**		−**0.11**	−**0.16:** −**0.06**	**<0.0001**
**Education: college**		**2.02**	**0.68: 3.36**	**0.004**
**BVMT T2**				
25 (OH)D (Sufficient Y/N)	0.34	−0.74	−2.01: 0.53	0.25
**Age**		−**0.16**	−**0.21:** −**0.11**	**<0.0001**
**Score leisure activities**		**2.75**	**0.06: 5.43**	**0.05**
**BVMT T3**				
25 (OH)D (Sufficient Y/N)	0.23	−0.73	−2.15: 0.69	0.31
**Age**		−**0.14**	−**0.20:** −**0.09**	**<0.0001**
BVMT-Total Score				
25 (OH)D (Sufficient Y/N)	0.248	−2.88	−7.23: 1.462	.189
EDSS		−3.23	−5.37:−1.09	0.004
**BVMT DR**				
**25 (OH)D (Sufficient Y/N)**	**0.31**	−**1.74**	−**3.01:** −**0.47**	**0.008**
**Age**		−**0.14**	−**0.20:** −**0.09**	**<0.0001**
**MOCA**				
**25 (OH)D (Sufficient Y/N)**	**0.16**	−**0.86**	−**2.27: 0.56**	**0.23**
**Age**		−**0.08**	−**0.14:** −**0.02**	**0.006**
**Anxiety score**		−**1.17**	−**2.27:** −**0.07**	**0.04**

Variables entered in all models are: 25 (OH)D (Sufficient Y/N), Disease duration, EDSS, Age, Education: college, Education: high school, Physical activity, smoking, alcohol, score leisure activities, Anxiety score at baseline, Depression score at baseline, Total Hopkins score at baseline. Abbreviations: MoCA = Montreal Cognitive Assessment; SDMT = Symbol Digit Modalities Test; BVMT-R = Brief Visuospatial Memory test.

**Table 4:** Multivariate analysis for the predictors of the 7 cognitive performance tests at 3 months (stepwise method).

Predictors	STROOP			
	R^2^	Unstandardized Beta	95% CI	P value
25 (OH)D (Sufficient Y/N)	0.26	2.06	−3.05: 7.17	0.42
**Anxiety score**		−**7.44**	−**11.46:** −**3.42**	**<0.0001**
**Age**		−**0.24**	−**0.45:** −**0.02**	**<0.0001**
**SDMT**				
25 (OH)D (Sufficient Y/N)	0.39	−5.79	−12.52: 0.94	0.09
**Education: college**		**14.54**	**6.18: 22.90**	**0.001**
**Age**		−**0.35**	−**0.65:** −**0.05**	**0.02**
**Alcohol**		**7.92**	**0.51: 15.32**	**0.04**
**BVMT T1**				
25 (OH)D (Sufficient Y/N)	0.21	0.01	−1.43: 1.45	0.99
**Alcohol**		**1.83**	**0.26: 3.41**	**0.02**
**EDSS**		−**0.99**	−**1.85:** −**0.14**	**0.02**
**BVMT T2**				
**25 (OH)D (Sufficient Y/N)**	**0.27**	−**1.69**	−**3.18:** −**0.20**	**0.03**
**Score leisure activities**		**4.90**	**1.89: 7.92**	**0.002**
**Age**		−**0.10**	−**0.17:** −**0.04**	**0.002**
**BVMT T3**				
25 (OH)D (Sufficient Y/N)	0.23	−0.73	−2.15: 0.69	0.31
**Age**		−**0.14**	−**0.20:** −**0.09**	**<0.0001**
BVMT-Total Score				
25 (OH)D (Sufficient Y/N)	0.48	−0.17	−4.57: 4.22	.936
EDSS		−2.55	−5.01:−0.093	0.042
Education: College		4.377	0.658: 8.09	
Score leisure activities		9.36	1.09: 17.637	0.022
Alcohol		4.78	0.63: 9.49	0.047
**MOCA**				
25 (OH)D (Sufficient Y/N)	0.31	−0.71	−2.22: 0.81	0.36
**Education: College**		**3.90**	**2.05: 5.76**	** < 0.0001**
**EDSS**		−**0.91**	−**1.79:** −**0.03**	**0.04**

Variables entered in all models are: 25 (OH)D (Sufficient Y/N), Disease duration, EDSS, Age, Education: college, Education: high school, Physical activity, smoking, alcohol, score leisure activities, Anxiety score at baseline, Depression score at baseline, Total Hopkins score at baseline. Abbreviations: MoCA = Montreal Cognitive Assessment; SDMT = Symbol Digit Modalities Test; BVMT-R = Brief Visuospatial Memory test.

**Table 5:** Cognitive fields measured by the cognitive tests battery.

Cognitive field	MoCA	Stroop	SDMT	BVMT-R
Verbal Memory	X			
Learning	X			X
Working Memory	X		X	
Interference				
Mental Flexibility		X		
Attention: Selective and Divided	X	X	X	
Information Processing Speed		X	X	
Visual Tracking	X		X	X
VisuoSpatial	X			X
Short Term Memory	X			X
Long Term Memory	X			X
Recognition	X			X
Language	X			
Abstraction	X			
Orientation	X			

MoCA = Montreal Cognitive Assessment; SDMT = Symbol Digit Modalities Test; BVMT-R = Brief Visuospatial Memory test.
