# Supplementary material for: Effect of Vitamin D Replacement on Cognition in Multiple Sclerosis Patients
Source: Sci Rep. 2017 Apr 4;7:45926. doi: 10.1038/srep45926 (PMC5379671; doi:10.1038/srep45926)
Supplement: Supplementary Information [file srep45926-s1.pdf]

Study Protocol

Effect of Vitamin D Replacement on Cognition in Multiple Sclerosis Patients

Hala Darwish, PhD[1, 2], Ribal Haddad MD[2], Sahar Osman, MD[2+], Stephanie Ghassan, MD[2+], Bassem Yamout, MD[2, 3], Hani Tamim, PhD[4] and Samia Khoury, MD[2, 3 *]

[1] American University of Beirut, Faculty of Medicine, Hariri School of Nursing, Beirut, Lebanon

[2] American University of Beirut Medical Center, Nehme and Therese Tohme Multiple Sclerosis Center, Beirut, Lebanon

[3] American University of Beirut, Faculty of Medicine, Neurology Department, Beirut, Lebanon

[4] American University of Beirut, Faculty of Medicine, Department of Internal Medicine and Clinical Research Institute, Beirut, Lebanon

[+] These authors contributed equally to this work

In this prospective observational cohort study that was conducted at the Multiple Sclerosis Center of the American University of Beirut Medical Center between the years 2012-2014. 111 MS patients met the study eligibility based on the following criteria:

Inclusion Criteria:

- Males and females aged 18 or older with Relapsing Remitting MS or Clinically Isolated Syndrome, diagnosed by one of the neurologists according to McDonald's 2010 criteria.
- Clinically stable with no relapses (defined as an episode of neurologic dysfunction lasting at least 24 hours) within four weeks of enrollment.
- On interferon beta with no gadolinium enhancing lesions on MRI and did not receive any corticosteroid therapy within four weeks prior to recruitment

Exclusion Criteria:

- Current use of drugs associated with an increased susceptibility to hypercalcemia

- Treatment with immune modulating/ suppressive drugs other than IFN-b within 6 weeks prior to enrolment
- Pregnancy
- Hypercalcemia
- eGRF<60
- History of primary hyperparathyroidism, hypercalcemia, renal dysfunction, cardiac disease, malignancy, or granulomatous disease.
- The occurrence of an exacerbation (defined as an episode of neurologic dysfunction lasting at least 24 hours) within 4 weeks of enrolment
- History of dementia or related disorders
- History of traumatic brain injury
- Diagnosis of epilepsy or history of seizure
- Diagnosis of psychiatric disease, substance abuse/dependence, alcohol abuse/dependence

After signing an informed consent, the research team (two trained research fellows) collected demographic data such as age and years of education, and lifestyle information such as smoking and exercise, using the *Brief Risk Factor Surveillance System (BRFSS).* Then, the *Hopkins Symptoms Checklist (HSCL-25)* was administered at baseline to screen all participants for any depressive or anxiety symptoms. The HSCL-25 was self-administered in Arabic language. This instrument is comprised of 25 items which are scored on a Likert scale from 1 (not at all) to 4 (extremely). Items 1 to 10 screen for anxiety symptoms, whereas items 11 to 25 screen for depressive symptoms. The mean of the total items represents the score on the total scale, and the means of the two subscales represent anxiety or depression symptoms. The research team notified the treating physicians when a participant had suicidal thoughts or score indicating severe depression. Suicidal thoughts were assessed by item 20 in the HSCL-25 which states “thoughts of ending your life”, and a score greater than 3.3 on the depression subscale indicated severe depression.

Cognitive assessment was then administered at baseline and at 3 months. The research fellows were trained on administering the tests at the psychological assessment center of the psychiatry department at the American University of Beirut. The psychometric specialist who is

an assistant professor of psychology, specialized in assessment, trained the research fellows. Their inter-rater reliability score was 0.90. The cognitive test battery was comprised of 4 tests, which are the Montreal Cognitive Assessment (MoCA), Symbol Digit Modalities Test (SDMT), Stroop test, and the Brief Visuospatial Memory test (BVMT-R). This test battery requires 45 minutes to be completed.

The MoCA is a rapid screening instrument for cognitive impairment in adults. It requires approximately 10 minutes to be administered and assesses different cognitive domains: attention and concentration, executive functions, memory, language, visuoconstructional skills, conceptual thinking, calculations, and orientation. The total possible score is 30 points and a score of 26 or above is considered as normal cognitive function. This test was administered in Arabic language. The test and administration instructions are available online for clinicians on http://www.mocatest.org/ . Attention, concentration and working memory are assessed using a sustained attention task (target detection using tapping), a serial subtraction task, and forward/backward digit span. Executive functions are assessed using an alternation task adapted from the trail-making B task, a phonemic fluency task, and a two-item verbal abstraction task. Short term memory is assessed through the short-term memory recall task which involves two learning trials of nouns and a delayed recall after approximately 5 minutes. Language is assessed using a three-item confrontation naming task with low-familiarity animals, repetition of two syntactically complex sentences, and the phonemic fluency task. Visuospatial abilities are assessed using a clock-drawing task and a three-dimensional cube copy. Finally, orientation to time and place is evaluated through asking about the date, place and city.

The SDMT assesses processing speed and requires 5 minutes of administration. The oral version was used. In this test, participants are given 90 seconds to substitute symbols with digits. The key with the 9 symbols and digits paired is given to the subject. Symbols tested upon are provided on the same sheet with the key. After explaining to the participant the test procedure, the examiner gives an example of symbol/digit substitution, then the participant is asked to train on 9 other pairings. The test starts following participants' successful symbol/digit pairing in the training phase. The score is the number of correctly coded items from 0-110 in 90 seconds.

The Stroop test which assesses attention was administered in Arabic and requires approximately 5 minutes to be completed. Participants are asked to read as many words as

possible from 3 stimulus sheets in 1 minute per sheet. Subjects are given a list of colors in black ink to read (Sheet 1), then a list of colors in their corresponding ink color (Sheet 2), and then finally a list of colors with incongruent ink colors (Sheet 3). The number of words read in a minute (minus the errors) was the dependent measure and interference was calculated. Four scores were generated, one score for each stimulus sheet and an interference score.

The BVMT-R assesses visuospatial memory and requires approximately 25 minutes to be administered. This test is composed of 5 trials. The first 3 are learning trials, followed by 1 delayed recall trial, and finally a recognition trial. In the first part, participants are shown a stimulus sheet comprised of 6 different geometric figures. In each learning trial, this sheet is shown for 10 seconds only and the participant is then asked to draw the figures as detailed as possible and in the right position on the answer sheet. After 25 minutes, subjects are asked to recall the figures again. Finally, in the recognition trial, participants are asked to identify which of 12 figures were included among the original geometric figures shown in the learning trials.

All subjects were handed a diary to document their daily ingestion of food known to be rich in vitamin D and to document their body parts exposure to sun as well. A research assistant contacted each participant weekly to collect information on his or her food intake and to ensure that participants are filling their diaries, which were collected at the end of the three months period.

Blood was drawn and serum 25(OH)D was analyzed at the Endocrine Core laboratory at the American University of Beirut Medical Center using Roche Diagnostics total assay. Based on their 25 OH(D) level results subjects were then assigned to sufficient (25(OH)D > 35 ng/ml) and deficient (<25ng/ml) 25(OH)D groups. 23 participants with 25(OH)D levels between 25 and 35 ng/ml were then excluded from the study. Subjects with deficient 25(OH)D levels were given a high dose vitamin D_3 supplementation (10,000 IU daily for 3 months). The research assistant contacted participants weekly to ensure compliance with their supplementation.

At 3 months, 22 subjects were lost to follow up, of the 66 who came back for a serum 25(OH)D analysis, 61 completed the same cognitive assessment and 30 completed and returned their food and sun exposure diaries.

Statistical Methods

Eighty-eight participants who met 25(OH) D eligibility criteria were included in the analyses of baseline characteristics. Descriptive statistical analysis was performed for the predictor variables 25(OH)D status (sufficient Y/N),– 25(OH)D, age, years of education, physical activity, sun exposure, dietary vitamin D intake, smoking, leisure activity and alcohol consumption.. Independent sample *t*-tests was conducted; missing cases were excluded from the analysis. Paired *t*-tests (before and after and between groups) and Pearson's r correlations were performed between predictor and outcome variables; MoCA, Stroop, BVMT-Trials 1 to 3, total score, BVMT-DR, and SDMT, on complete cases only. A stepwise linear regression analysis was then conducted to examine the relationship between predictor and outcome variables at baseline and at 3 months.

SPSS Statistics for Windows, version 22 (IBM Corp., Armonk, N.Y., USA) was used for all statistical analyses.
